# Benchmarking Reconstructive Spectrometer with Multiresonant Cavities

**DOI:** 10.1021/acsphotonics.4c00915

**Published:** 2024-08-15

**Authors:** Chunhui Yao, Kangning Xu, Tianhua Lin, Jie Ma, Chumeng Yao, Peng Bao, Zhitian Shi, Richard Penty, Qixiang Cheng

**Affiliations:** † Centre for Photonic Systems, Electrical Engineering Division, Department of Engineering, University of Cambridge, Cambridge CB3 0FA, U.K.; ‡ GlitterinTech Limited, Xuzhou 221000, China

**Keywords:** reconstructive spectrometer, multiresonant cavity, compressive sensing, mutual correlation coefficient, spectral-pixel-to-channel ratio

## Abstract

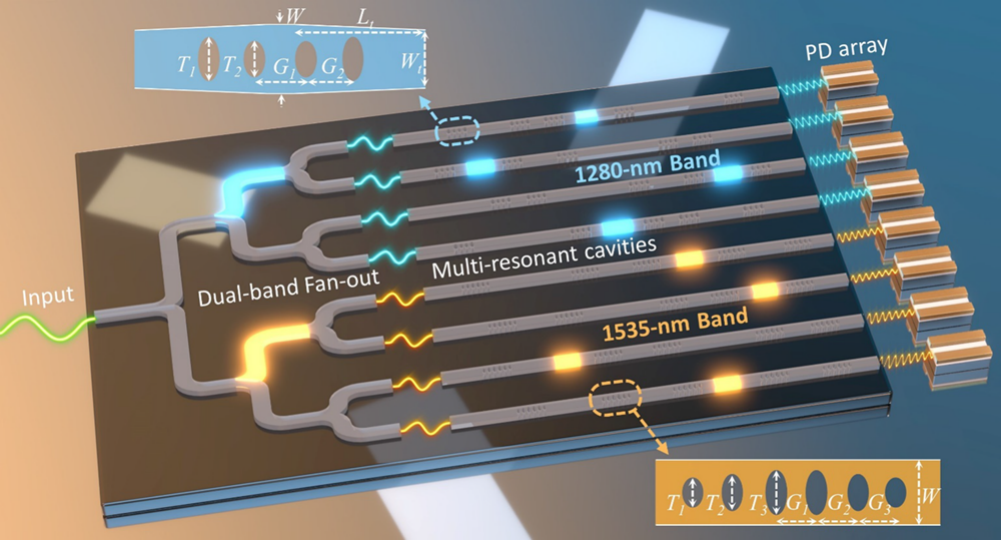

Recent years have seen the rapid development of miniaturized reconstructive spectrometers (RSs), yet they still confront a range of technical challenges, such as bandwidth/resolution ratio, sensing speed, and/or power efficiency. Reported RS designs often suffer from insufficient decorrelation between sampling channels, which, in essence, is due to inadequate engineering of sampling responses. This in turn results in poor spectral-pixel-to-channel ratios (SPCRs), typically restricted at single digits. So far, there lacks a general guideline for manipulating RS sampling responses for the effectiveness of spectral information acquisition. In this study, we shed light on a fundamental parameter from the compressive sensing (CS) theory—the average mutual correlation coefficient *ν*—and provide insight into how it serves as a critical benchmark in RS design. To this end, we propose a novel RS design with multiresonant cavities, consisting of a series of partial reflective interfaces. Such multicavity configuration allows the superlative optimization of sampling matrices to achieve minimized *ν*. Experimentally, we implement a single-shot, dual-band RS on a SiN platform, realizing an overall operation bandwidth of 270 nm and a <0.5 nm resolution with only 15 sampling channels per band. This leads to a record high SPCR of 18.0. Moreover, the proposed multicavity design can be readily adapted to various photonic platforms, ranging from optical fibers to free-space optics. For instance, we showcase that by employing multilayer coatings, an ultrabroadband RS can be optimized to exhibit a 700 nm bandwidth with an SPCR of over 100.

## Introduction

The burgeoning market for in situ, in vivo, and in vitro optical spectroscopic applications—ranging from wearable healthcare monitoring to compact optical imaging systems—has catalyzed the rapid development of miniaturized spectrometers.^1−3^ However, the miniaturization of spectrometers inevitably compromises their performance specifications such as bandwidth and/or resolution. Meanwhile, the evolving application landscape also prioritizes other metrics, such as cost, sensing speed, and power efficiency.^4,5^ For example, the quick detection of explosives or chemical threats calls for portable, battery-operated spectrometers that features nanometer-scale resolution.^6^ These multifaceted demands are even more pronounced for the spectroscopic sensors embedded in smartphones or Internet-of-Things (IoT) devices.^7,8^ Furthermore, many NIR or MIR spectroscopy applications, including the biomedical sensing of urea or glucose^9,10^ or the industrial detection of fuel,^11^ necessitate the identification of signature spectral peaks across various wavelength bands. Fulfilling all of the above-mentioned criteria presents a significant technical bottleneck.

In recent years, reconstructive spectroscopy (RSs) has emerged as a transformative paradigm in the field. Unlike traditional demultiplexing-to-detection spectrometers that rely on dispersive elements or narrowband filters to linearly decompose the incident light,^12,13^ RSs spatially or temporally sample the entire incident spectrum, resolving a quantity of spectral pixels via compressive sensing (CS) theory and regression algorithms. The spectral-pixel-to-channel ratio (SPCR), also termed the reconstructive compression ratio, signifies the ability of RSs to accommodate the largest number of spectral pixels with the fewest sampling channels.

**Figure 1:**
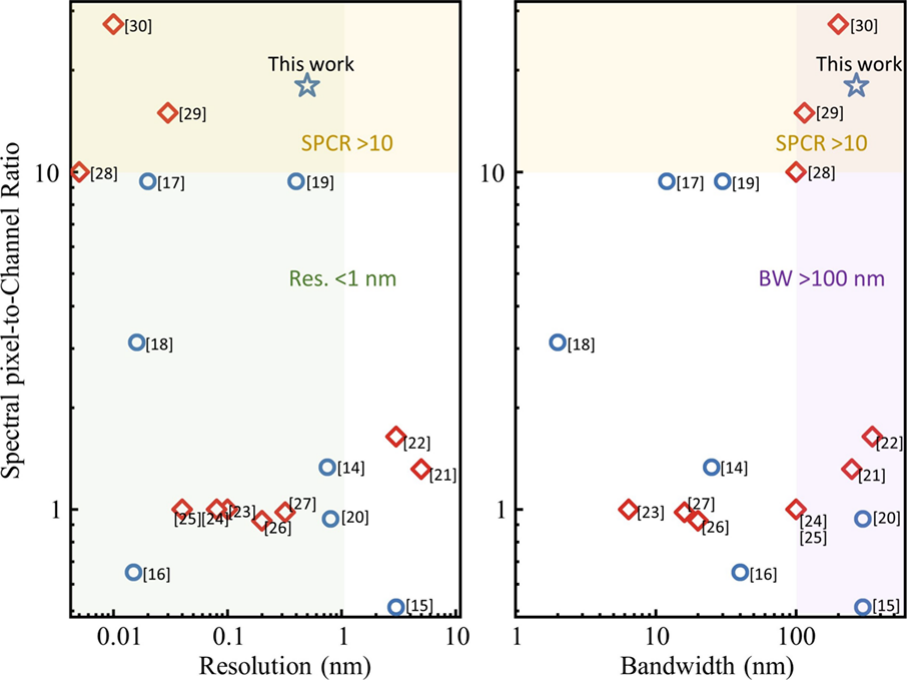
Performance comparison of various state-of-the-art RSs in terms of the spectral pixel-to-channel ratio (SPCR), resolution, and bandwidth. The red diamonds and blue circles represent the active and passive RSs, respectively. The colored regions highlight the achievement of our device as it simultaneously attains <1 nm resolution, >100 nm bandwidth, and >10 SPCR.

Figure 1 summarizes the SPCRs for the state-of-the-art RS designs, along with their resolutions and bandwidths.^14−30^ Here, we generally divide them into two categories based on their operation mechanism: active RSs and passive RSs. The former is exemplified by those with MEMS,^22^ tunable microrings,^24^ and reconfigurable or programmable photonic integrated circuits.^29,30^ By temporally tuning hundreds or even thousands of sampling responses, these devices have demonstrated outstanding resolutions, reaching the scale of picometers. Nevertheless, the active scheme inevitably leads to increased power consumption and considerable sensing times. Also, these temporal channels often suffer from poor decorrelations due to the limited phase-intensity modulation range, leading to inefficiencies and redundancies in the sampling process. This shortcoming is clearly illustrated in Figure 1, where most active RSs exhibit low signal-digit SPCRs around one. In contrast, passive RSs take advantage of single-shot measurement and enjoy the freedom of manipulating each channel response individually. Nevertheless, the spatially split incident light restricts their channel counts, typically ranging from a few to several dozens. In addition, most current passive designs are limited by lumped filtering structures, such as disordered scattering media,^14^ quantum dots,^15^ or metasurface,^20^ facing difficulties in fully engineering the channel responses. As a result, their SPCRs are still confined to single digits or even lower.

In this paper, we draw a general guideline for RS design from the basics of CS theory, highlighting that the average mutual correlation coefficient *ν* of sampling matrices serves as a critical benchmark that adversely correlates to both the SPCR and reconstruction accuracy. Meanwhile, we propose a novel RS design scheme that utilizes multiresonant cavities, composing of cascaded interfaces with partial reflectance. Such a multicavity system features an extensive parameter space, enabling flexible engineering of its spectral response over a broad bandwidth. Thereby, simply by adjusting the cavity lengths and reflectance, a multiresonant cavity system can be systematically optimized to establish sampling matrices with minimized *ν*. As a proof-of-concept demonstration, such a multicavity design is implemented on the silicon nitride (SiN) integration platform to create a single-shot, dual-band RS with photonic crystal nanobeams. Here, aligned with the spectroscopic sensing needs for biomarkers such as lactate and glucose, we strategically select two wavelength bands centered around 1280 and 1535 nm as observation windows.^31−33^ Two sets of photonic crystal nanobeams, customized as broadband, ultralow loss, partial reflective waveguide mirrors, are cascaded to form the multiresonant cavities, each occupying a footprint of less than 200 μm^2^. Experimentally, our device achieves a total operation bandwidth of 270 nm (ranging between 1227 and 1334 nm and 1453–1616 nm, respectively) and a <0.5 nm resolution, using only 15 sampling channels per band. This yields an SPCR of 18.0, which, to the best of our knowledge, is a new record for passive miniaturized spectrometers (as shown by Figure 1). The SiN platform also offers an excellent thermal stability of over ±5.0 °C. Moreover, the proposed RS design can be seamlessly applied to various optical platforms, including photonic integrated circuits,^34^ grating-based optical fibers,^35^ multilayered optical coating systems,^36^ and free-space optics.^37^ As an illustration, we demonstrate that based on multilayer coatings, an ultrabroadband RS can be readily constructed to feature a 700 nm bandwidth and 0.04 nm resolution, leading to a SPCR of 136.7. In summary, this study points out a promising direction toward compact, high-performance RS with great scalability and robustness.

## Results

### Design and Optimization

**Figure 2:**
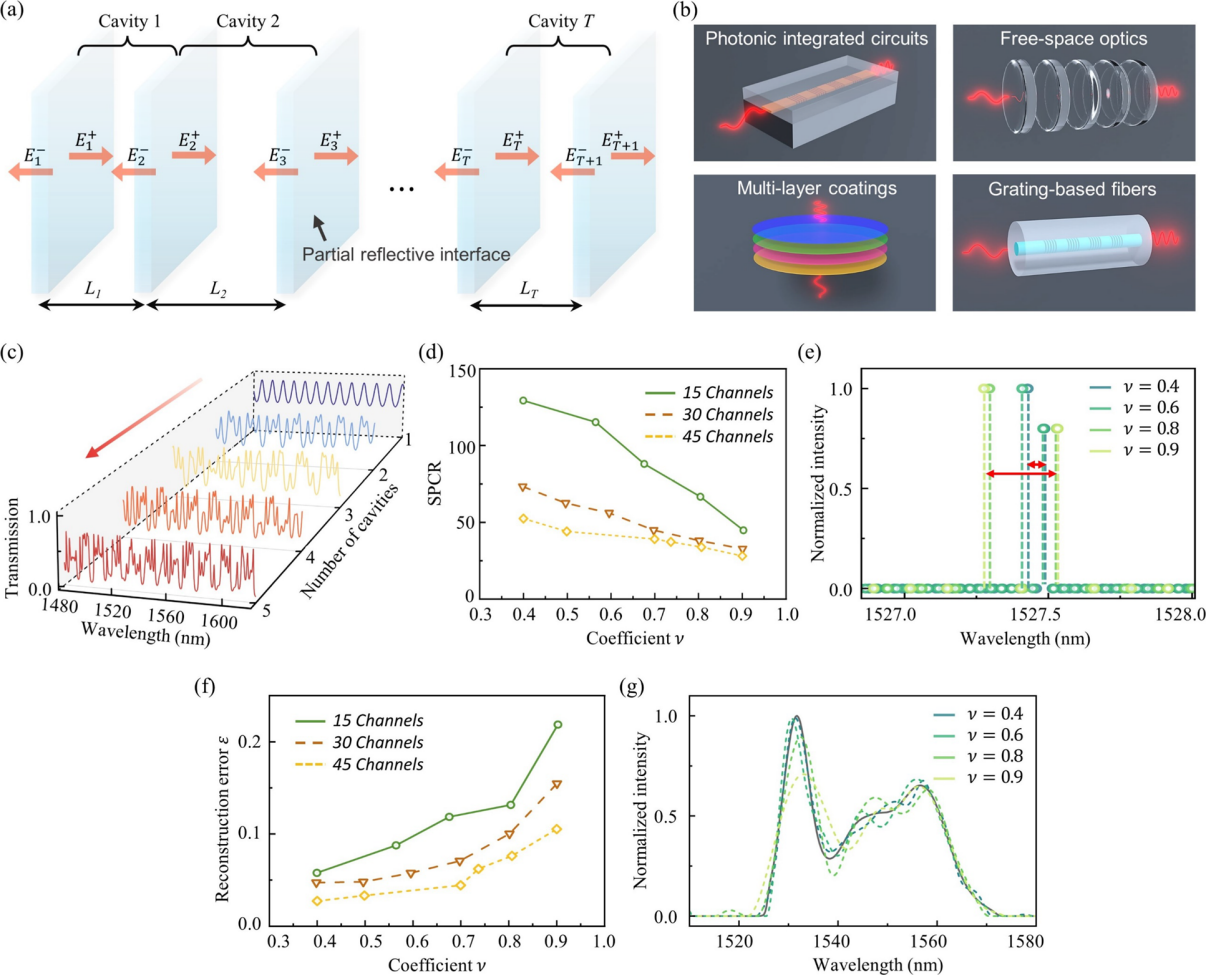
Design and optimization of multiresonant cavities. (a) Schematic of the proposed multiresonant cavities with cascaded partial reflective interfaces. (b) Conceptual illustrations of the proposed multicavity scheme implemented on various photonic platforms. (c) Simulated transmission spectra of multiresonant cavities with different cavity numbers. (d) Simulated relationship between the spectrometer SPCR and coefficient *ν*. (e) Reconstructed dual-peak signals with minimum resolvable spectral spacings using 15-channel sampling matrices with different values of *ν*. (f) Simulated relationship between the reconstruction error and coefficient *ν*. (g) Reconstructed spectra for resolving a broadband continuous signal using 15-channel sampling matrices with different values of *ν*, exhibiting different levels of accuracy.

Figure 2a shows the schematic of the core element in our proposed RS design: the multiresonant cavity. Each multicavity consists of a sequence of partial reflective interfaces positioned at varying spacings, thus offering a distinctive spectral response. By arranging various multicavity channels for parallel sampling, a single-shot RS can be assembled. Notably, the desired partial reflective interfaces can be produced by using different mature fabrication techniques. Figure 2b shows the conceptual diagrams of the proposed multicavity configuration implemented on various platforms. Detailed elaborations regarding these implementation schemes can be found in the Discussion section.

To describe light propagation within the multiresonant cavity, we utilize the transfer matrix method.^38^ As denoted in Figure 2a, the electrical field amplitudes in the *i*th cavity adhere to the following set of equations, as^39^
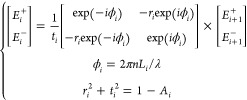
1where *E*_*i*_^+^, *E*_*i*_^–^, *E*_*i*+1_^+^, and *E*_*i*+1_^–^ denote the forward and backward propagating electric-field vectors at the boundaries of interface *i* and *i* + 1, respectively. *r_i_* and *t_i_* are the amplitude reflection and transmission coefficients for interface *i*, respectively. *nL*_*i*_ represents the effective optical path length of the *i*th cavity (i.e., the product of the filling material’s refractive index *n* and the spacing between adjacent pairs of interfaces), and *A*_*i*_ accounts for the overall optical loss within the cavity. As such, for a multicavity system with *T* cavities (i.e., *T* + 1 interfaces), eq 1 further extends to
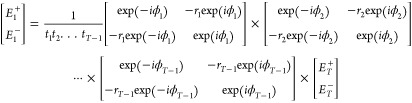
2a
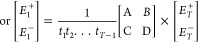
2bwhere *A*, *B*, *C*, and *D* are the coefficients of the resulting matrix. Accordingly, the amplitude transmission at wavelength *λ* is
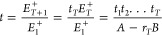
3

Based on eq 3, the transmission spectrum of a multicavity channel (i.e., its spectral response) can be calculated. For example, Figure 2c illustrates how transmission spectra evolve as the number of cavities increases (with the reflectance set at 0.15). It can be seen that with more than three cavities, the output spectra lose periodic patterns and exhibit denser spectral fluctuations and increasing spectral randomness. Notably, such multicavity systems do not require stringent resonance conditions to produce pseudorandom spectral responses, thereby allowing for relaxed tolerance to fabrication errors.

Mathematically, when an unknown incident spectrum *Φ*(*λ*) traverses such a multiresonant cavity, the resultant output power intensity *I* is the integral of *Φ*(*λ*) and the channel response, denoted as *S*(*λ*), over the wavelength. Likewise, for *M* multicavity channels, the corresponding output power intensities *I*_*M*×1_ can be discretized and expressed in a matrix format,^4^ as
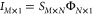
4where *N* represents the number of spectral pixels in the wavelength domain and *S_M__×N_* is the sampling matrix. The ratio of *N* to *M* represents the spectrometer’s SPCR. Previous research in RSs has broadly suggested that achieving a high SPCR necessitates a sampling matrix with distinct channel responses for efficient and uncorrelated sampling.^5^ However, there has been a notable absence of a quantifiable benchmark to assess the performance of these sampling matrices. In this work, we bridge this gap using the fundamental principle of CS, which is a unique sampling technique that enables unambiguous reconstruction of the original signal from a set of overall sampled data. This approach allows the sampling rate to be significantly lower than the traditional Nyquist rate. Specifically, when applied to spectrum reconstruction, the CS algorithms aim to find the orthonormal basis *Ψ* to ensure that the incident spectrum *Φ*(*λ*) can be expressed as
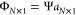
5where *a_N×1_* is a sparse vector with k nonzero elements (*k* ≪ *N*). Consequently, eq 4 can be rewritten as
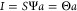
6

According to the CS theory, the minimum number of sampling channels (i.e., *M*) required to unambiguously reconstruct a *N*-dimension incident signal follows:^40^
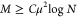
7where *μ* represents the mutual coherent coefficient of the product of the sampling matrix and the orthonormal basis *Ψ*, and *C* is a constant related to the level of sparsity *k* and the performance of reconstruction algorithms. The mutual correlation coefficient *μ* is defined as
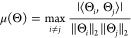
8where Θ_*i*_ and Θ_*j*_ are the *i*th and *j*th column vectors of matrix Θ, respectively. In practice, since the spectral responses generated from physical photonic structures are naturally continuous, the coefficient *μ* should be represented by the average mutual coherence coefficient *ν* as it reflects the overall reconstruction ability over the working bandwidth, which is written as^41^
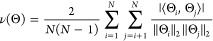
9

Please find more detailed definitions of *μ* and *ν* in the Supporting Information Section 1. According to eq 7, it is evident that with a fixed channel number *M*, a smaller value of *μ/ν* implies a stronger reconstruction capability of the spectrometer, that is, higher SPCR. Note that since the derivation of eq 7 is tied to the characteristics of reconstruction algorithms,^41^ in this study, we consistently employ a standard convex optimization algorithm to solve the inverse problem defined by eq 4.^42^ Its process can be written as
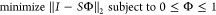
10

To enhance the noise tolerance during the reconstruction of continuous signals, an additional regulation term can be further introduced to eq 10, as
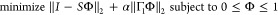
11where *α* denotes a weight coefficient that can be optimized via cross-validation analysis and *Γ*_1_ is a matrix used to compute the first derivative of *Φ*.

The proposed RS scheme with multiresonant cavities offers a rich parameter space for system-level optimization. To exploit this, we simulate a series of multicavity channels via eq 3, sweeping the key parameters—the effective optical path lengths of cavities and the interface reflectance—to establish sampling matrices with diverse channel numbers and *ν* values. We set the cavity number at five and limit the cavity’s largest effective optical length below 100 μm as a balanced choice to ensure both the high sampling performance and compact device footprint. The particle swarm optimization (PSO) algorithm is employed to search for the optimal parameter configurations of multicavities, with the aim of achieving different *ν* levels.^43,44^ As a result, various transmission matrices with *ν* ranging between around 0.4–0.9 are generated. Note that these simulations assume an ideal scenario where the loss of interfaces and dispersion effect are neglected, such that the actual *ν* values are expected to be higher.

Our investigation then focuses on the relationship between the SPCR and *ν*, as shown by Figure 2d. Here, we determine the spectrometer’s resolution and SPCR by resolving dual-peak laser signals with different spectral spacings over a broad bandwidth (i.e., following the Raileigh’s criteria). For instance, Figure 2e reveals that as ν decreases from 0.9 to 0.4, the minimum resolvable spectral spacing (i.e., the resolution) for an RS with 15 sampling channels reduces by nearly 3-fold. The clear downward trend in Figure 2d further confirms that a lower *ν* value corresponds to a higher SPCR, aligning well with the CS theory as presented by eq 7. Meanwhile, we also explore the impact of *ν* on the reconstruction accuracy by solving continuous broadband signals. The reconstruction results are assessed using the L2-norm relative error:
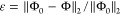
12where *Φ* is the reconstructed spectrum and *Φ*_0_ is reference. As illustrated by Figure 2f, a smaller *ν* also effectively reduces the reconstruction errors. Figure 2g provides a vivid example of this trend by depicting the retrieval of an ASE spectrum from an erbium-doped fiber amplifier (EDFA), showing the adverse relationship between the value of *ν* and reconstruction accuracy. It should be noted that all the above findings are based on statistical analysis, achieved by repeatedly reconstructing different incident spectra and then averaging the results. Hence, while a transmission matrix with a lower *ν* might not always be the most advantageous for a specific spectrum, it generally has a greater probability of yielding a higher resolution and accuracy. For more details and discussions about these simulations, please refer to Supporting Information Section 2.

### Device Implementation and Simulation

**Figure 3:**
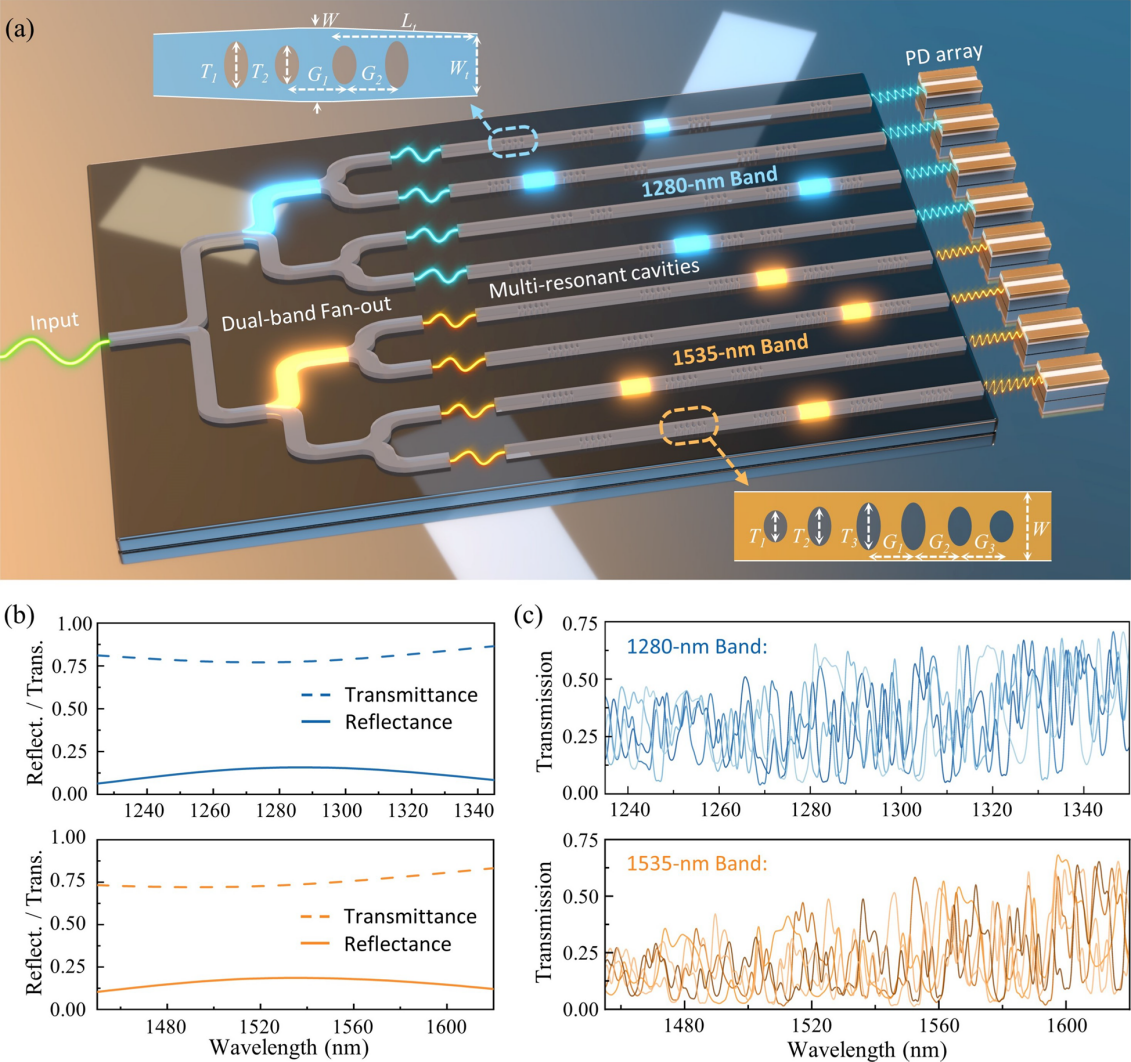
Design and simulation of single-shot dual-band RS. (a) Conceptual schematic of our dual-band spectrometer design based on multiresonant cavities. PD: Photodetector. The insets show the schematics of nanobeam waveguide mirrors optimized for the two operational bands, respectively. (b) FDTD-simulated transmittance and reflectance of the two nanobeam mirrors, respectively. (c) FDTD-simulated spectral responses of several exemplary channels of the two bands, respectively.

Figure 3a shows the schematic of our single-shot dual-band RS design based on photonic integrated circuits. Two sets of multicavity channels with customized photonic crystal nanobeams are developed at the spectral windows around 1280 and 1535 nm. For clarity, we refer to these two bands as the 1280 nm band and 1535 nm band, respectively. An ultrabroadband Y-splitter developed via the inverse design method is allocated in front of all the sampling channels to combine their working bandwidths.^45^ Its detailed design procedures and measured transmission characteristics are shown in Figure S1 in the Supporting Information Section 3.

We cascade six nanobeam waveguide mirrors per sampling channel and systematically optimize their cavity lengths to achieve a low *ν*. Each nanobeam mirror features multiple elliptical etching holes, serving as a low-loss broadband interface with desired reflectance.^46,47^ The insets in Figure 3a schematically show the nanobeams optimized for respective wavelength bands. The hole numbers are selected to be 4 and 6, respectively. *T*_*i*_ (i = 1, 2, or 3) denotes the lengths of the major axes of the elliptical holes, while the minor axis of all these holes is consistently set at 250 nm, in line with the fabrication feature size. *G*_*i*_ (i = 1, 2, or 3) represents the distance between adjacent holes. *W* defines the waveguide width. Note that the 1280 nm band nanobeam mirror includes a pair of symmetrical tapers to further reduce the loss, where *L*_*t*_ and *W*_*t*_ represent the taper length and tip width, respectively. PSO algorithm is also employed to optimize these geometric parameters, targeting to enable the broadest possible working bandwidth. The parameter details are provided in Table S1 in the Supporting Information Section 4. Figure 3b shows the finite-difference time-domain (FDTD) simulated transmittance and reflectance of the nanobeam mirrors for the two wavelength bands, respectively. As can be seen, the mirrors exhibit consistent reflectance of about 0.12 and 0.15 over the wavelength ranges from 1225 to 1345 and 1450–1620 nm, respectively, both with insertion losses of around 0.3 dB. As such, the whole transmission matrices for the two respective bands are simulated. Figure 3c displays a few exemplary channel spectral responses. The calculated *ν* values for these two matrices are 0.75 and 0.73, respectively. The main barrier to further minimizing *ν* is imposed by the fabrication feature size. For example, our simulation results indicate that reducing the mirror reflectance to below 0.08 could effectively lower the *ν* value to <0.5. However, maintaining a low and consistent reflectance over a wide bandwidth is challenging with the limitation of fabrication feature size at 250 nm. Thus, we slightly trade-off the value of *ν* for a larger bandwidth.

### Experimental Testing

**Figure 4:**
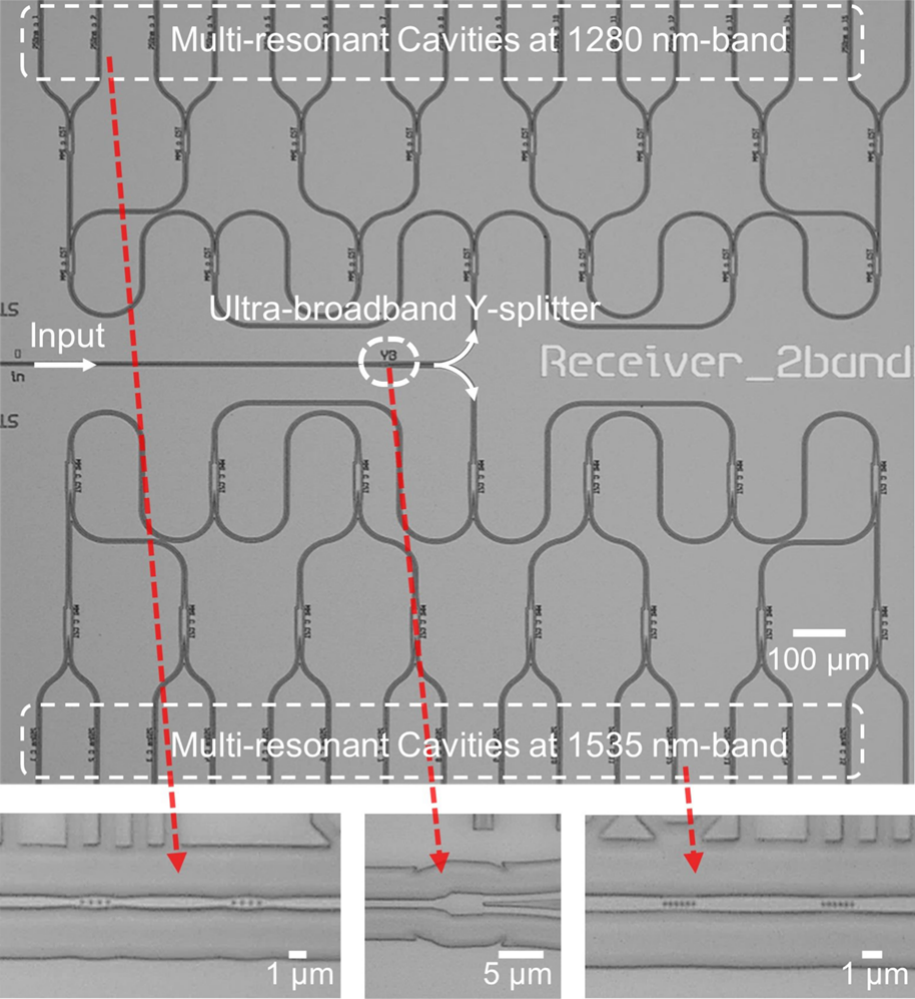
Microscope image of the fabricated spectrometer on a SiN platform. The insets show the enlarged views of the ultrabroadband Y-splitter and the nanobeam mirrors for each wavelength band, respectively.

Figure 4 presents the microscope image of the fabricated RS. The inset depicts the enlarged views of the ultrabroadband Y-splitter and the nanobeam mirrors, respectively. The spectrometer chip is fabricated via a CORNERSTONE SiN multiproject wafer (MPW) run using standard DUV lithography, featuring a 300 nm thick LPCVD SiN layer sandwiched by a 3 μm buried oxide layer and a 2 μm silicon dioxide top cladding layer. The fabrication process involves two sequential etching steps: the first one to define the waveguides and associated components and the second specifically to pattern the photonic crystal nanobeams. The chip is placed on top of a thermoelectric cooler (TEC) for temperature stabilization during the testing.

**Figure 5:**
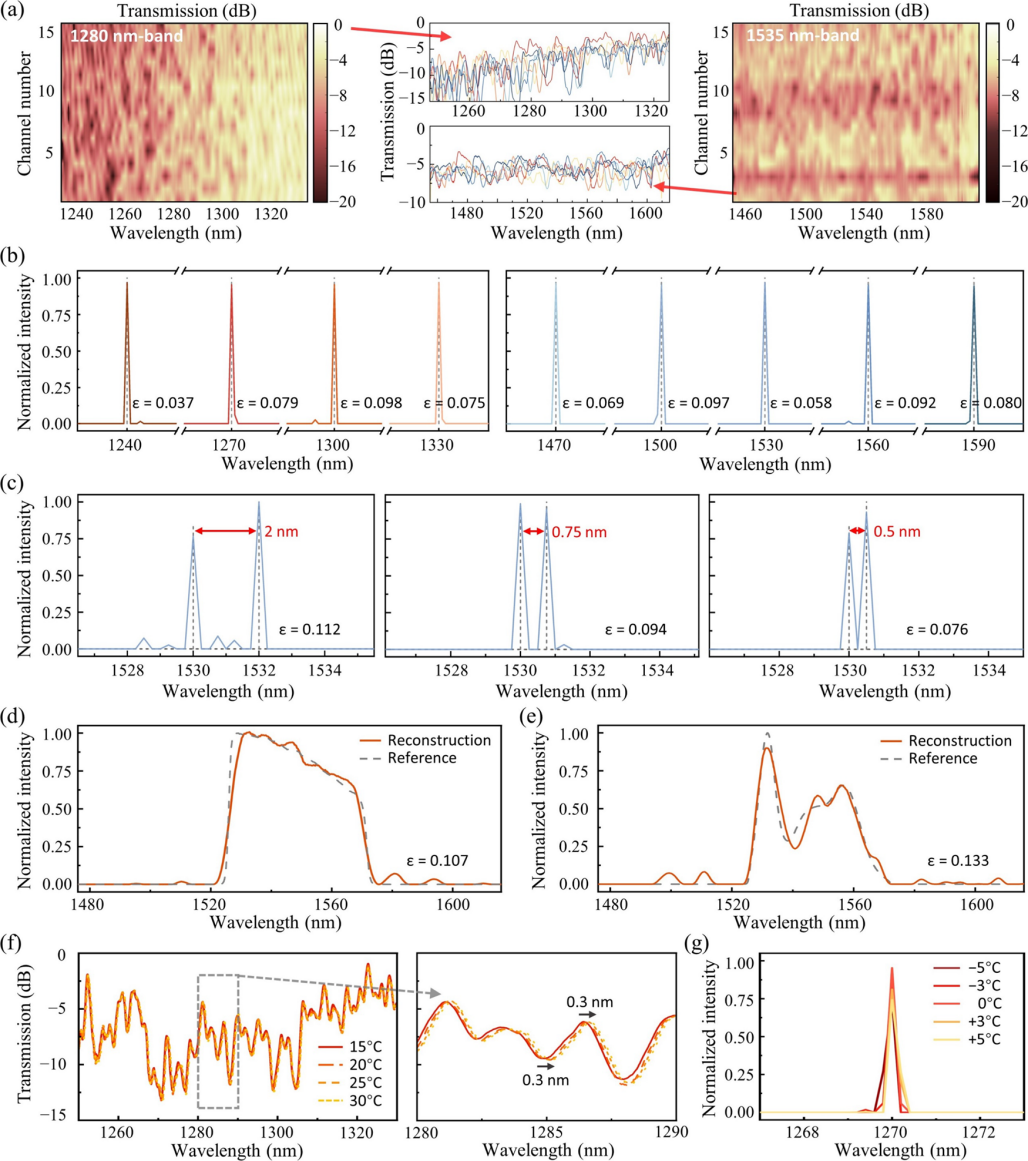
Spectrometer calibration and testing. (a) Normalized transmission matrices for the two operational bands, each with 15 sampling channels. The insets plot a few channel responses as examples. (b) Reconstructed spectra for a series of single laser peaks. The black dashed lines mark their center wavelengths. (c) Reconstructed spectra for dual laser peaks with a decreasing spectral spacing from 2 to 0.5 nm, respectively. (d,e) Reconstructed results for bandpass-filtered ASE spectra from an SOA and EDFA, respectively. (f) Measured transmission spectra of one specific sampling channel at different temperatures. The inset highlights a 0.3 nm redshift in spectral response when the temperature increases from 15 to 30 °C. (g) Reconstructed spectra for a single laser peak at different measurement temperatures, using the same transmission matrix precalibrated at 20 °C.

We calibrate the sampling matrices by launching the amplified spontaneous emission (ASE) spectra from two super luminescent diodes (SLDs), each covering a wavelength band, and then measure the channel transmission spectra by using a commercial spectrum analyzer (YOKOGAWA AQ6370D). Figure 5a shows the transmission matrices measured at the two wavelength bands, respectively. Here, the calibrations are conducted within a 107 nm spectral window from 1227 to 1334 nm and a 163 nm spectral window from 1453 to 1616 nm, respectively, limited by the bandwidth of the ASE sources—the actual spectrometer bandwidths are expected to be slightly larger. The measured channel insertion losses are approximately 3 dB for both bands, which can be attributed to insertion losses from the nanobeams and power splitting components. Accordingly, our spectrum reconstructions are performed within such 107 or 163 nm spectral ranges. The inset in Figure 5a reveals several representative sampling responses, showing the intensive spectral fluctuations induced by the multicavity system. Note that here the calibration wavelength range is limited by the bandwidth of the ASE sources, and the actual bandwidth of the spectrometer is expected to be larger. The coefficients *ν* of the two measured transmission matrices are both around 0.8, showing a minor degradation from our simulation outcomes. This discrepancy can be attributed to the distortions in channel spectral responses due to fabrication imperfections. Fortunately, the following experimental findings suggest that the spectrometer’s resolution and reconstruction accuracy are still maintained at a satisfactory level.

First, we launch a series of laser peaks at different wavelengths as narrowband signal inputs (using the Keysight 81609A tunable lasers) and record the corresponding output powers at each sampling channel. The reconstructions of these laser signals follow eq 10 using a convex optimization algorithm.^42^Figure 5b plots the resolved laser peaks at the two bands, with the full-width-half-maximums (FWHMs) consistently maintained at around 0.2 and 0.25 nm, respectively. The calculated relative errors *ε* of these laser signals range between 0.037 and 0.098. We also demonstrate the reconstruction of dual-peak laser signals to verify spectrometer resolution, as well as continuous, broadband spectra. Note that due to the limited light sources in our laboratory, this part of testing is only conducted on the 1535 nm band. Figure 5c shows the reconstructed dual-peak signals with a spectral spacing decreasing from 2 down to 0.5 nm. The well distinguished peak locations and intensities illustrate a resolution of less than 0.5 nm. Note that the resolution at 1280 nm band is anticipated to be even finer, as evidenced by the narrower FWHMs of the resolved single laser peaks. Moreover, we introduce the ASE spectra from a semiconductor optical amplifier (SOA) and an EDFA as continuous, broadband signals and resolve them in accordance with eq 11, as shown by Figure 5d,e. The respective reconstruction errors *ε* are 0.107 and 0.133.

The low thermo-optic effect of the SiN platform offers our device superior temperature tolerance. As shown by Figure 5f, the channel spectral responses only redshift around 0.3 nm when the temperature rises from 15 to 30 °C, (i.e., about 0.02 nm shifting per degree), while the waveform shape remains almost unaffected. For further quantification, we reconstruct a laser signal using the sampling matrix precalibrated at 20 °C, along with the output channel power intensities measured at different temperature settings, as shown by Figure 5g. The results reveal that the input laser peak can still be well resolved with a temperature variation of ±5 °C, illustrating a superior temperature stability of our device. In practice, on-chip temperature stabilization techniques and real-time temperature compensation algorithms can be utilized to help further escalate the spectrometer’s thermal robustness.^48^

Finally, to highlight the advances of our spectrometer across various performance metrics, we provide a comprehensive comparison against other competitors, as listed in Table 1. In addition to the highest SPCR, our dual-band device also stands out with the best temperature tolerance with the largest operation bandwidth. Note that in our fabricated RS, all of the edge couplers are spaced at a 250 μm pitch specifically to enable easy parallel alignment with our fiber array, which, however, enlarges the overall footprint. The actual footprint of a multiresonant cavity itself is less than 1 × 200 μm^2^. Also, this size can be further reduced when adapted to other integration platforms with a higher index contrast such as the silicon-on-isolator (SOI) platform.

**Table 1:** Performance Comparison with the State-of-the-Art Competitors

spectrometer	resolution	bandwidth	physical channel	spectral pixel-to-channel ratio	platform	footprint	temperature tolerance
disordered media^14^	0.75 nm	25 nm	25	1.3	silicon	50 × 25 μm^2^	±4.0 °C (simulation)
disordered media^55^	3 nm	40 nm	13	1.02	polymer	200 × 50 μm^2^	N.M.^a^
disordered media + mode decomposer^19^	0.4 nm	30 nm	8	9.4	polymer	30 × 12.8 μm^2^	N.M.
multimode waveguide^18^	0.01 nm	2 nm	40	5	silicon	500 × 500 μm^2^	±0.16 °C (simulation)
coherent network^17^	0.02 nm	12 nm	64	9.4	silicon	520 × 220 μm^2^	N.M.
2D microring lattice^16^	0.015 nm	40 nm	4096	0.65	silicon	1000 × 1000 μm^2^	N.M.
multiresonant cavity (this work)	<0.5 nm	270 nm	15 × 2	18.0	silicon nitride	<1 × 200 μm^2^ per cavity	±5.0 °C

a N.M.: Not mentioned.

## Discussion

Despite the *ν* value being compromised by both limited feature size and variations in fabrication, our single-shot dual-band RS still demonstrates a spectral pixel count of 326 and 214 for the two respective bands while maintaining the reconstruction relative errors around 0.1. This leads to a record-breaking SPCR of 18.0 in average. It is foreseeable that its performance can be further enhanced with advanced fabrication techniques. Besides photonic integrations, the proposed multicavity scheme can be adapted to other well-established photonic platforms to exploit their respective advantages. For example, in the realm of thin-film optics, commonly used optical coating materials, including dielectric materials such as titanium dioxide (TiO2) and silicon dioxide (SiO2), can be employed to effectively produce ultrabroadband reflective interfaces spanning from the ultraviolet all the way to far-infrared spectrum.^36,49,50^ Hence, the multiresonant cavity can be directly assembled by the coating of a sequence of such interfaces onto a single substrate. Similarly, fiber Brag gratings, particularly the long period gratings (LPGs) featuring wide working bandwidths, or the free-space optical microlens can be cascaded to create the desired multicavity scheme, both of which can be cost-effective and small footprint.^51−53^ Furthermore, microelectro-mechanical systems (MEMS) can be leveraged in tandem with the optical lenses to actively adjust the cavity spacings for the temporal tuning of sampling responses, making active but resonant RSs that occupy only one physical channel.^37,54^

**Figure 6:**
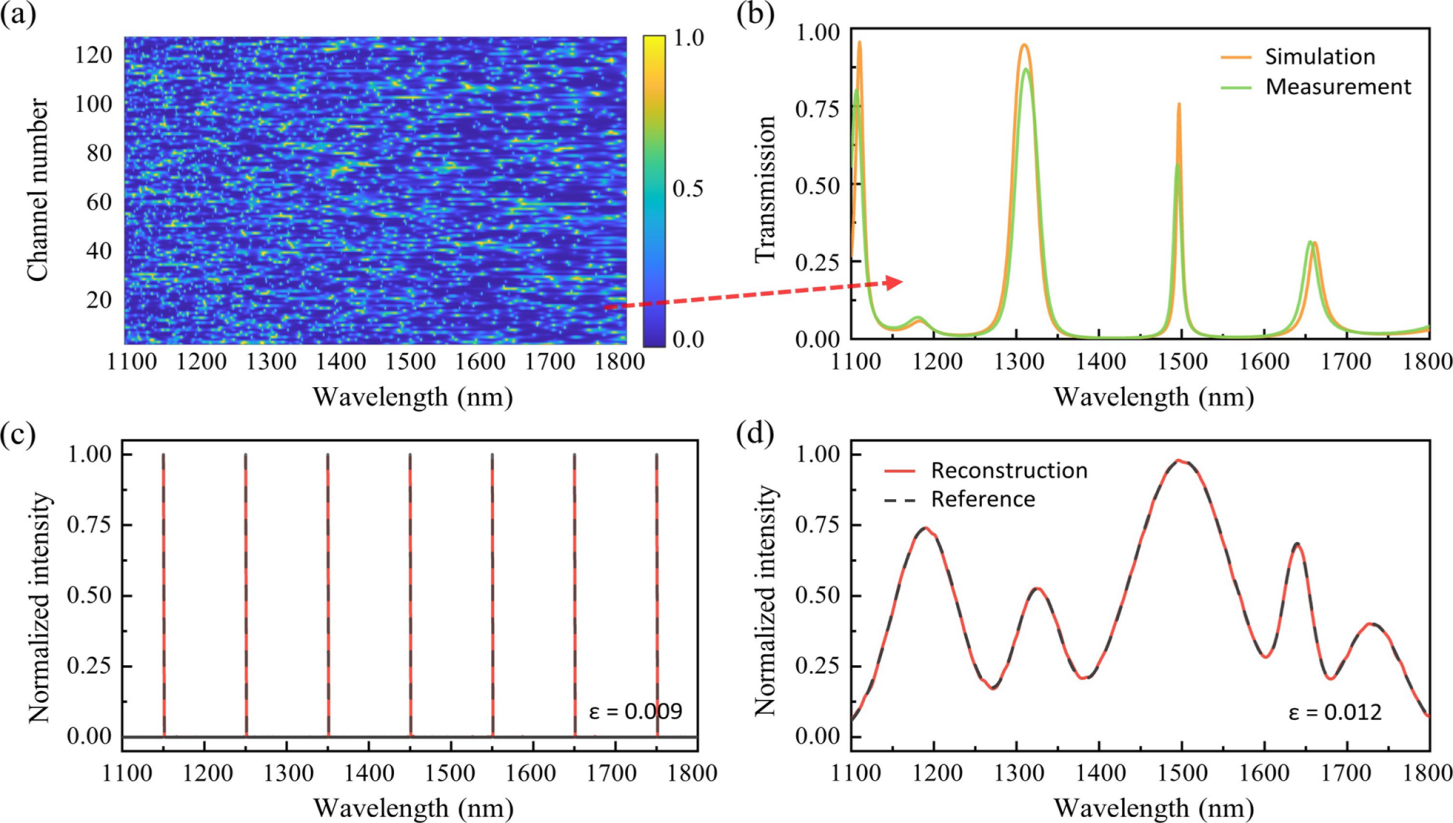
Exploration toward ultrabroadband free-space RS based on multilayer optical coatings. (a) Simulated 128-channel sampling matrix with the *ν* value optimized to only 0.38. (b) Simulated and measured transmission spectra of a specific sampling channel, showing a high degree of agreement. (c,d) Simulated reconstructed spectra for sparse and continuous incident signals, respectively.

Here, we preliminarily exploit the development of multilayered RSs using standard coating processes. As an example, we looked into a 60-layer alternating coating design of TiO2 and SiO2 on a glass substrate. By fine-tuning the thickness of each coating layer, we manage to fully engineer the overlaid transmission spectrum, achieving an ultrabroad bandwidth of 700 nm (from 1100 to 1800 nm). More design details can be found in Supporting Information, Section 5. Via a global optimization of layer thickness combinations, a 128-channel sampling matrix is created with the *ν* value optimized to only 0.38, as shown by Figure 6a. Using an evaporation coating machine, we fabricate one of the sampling channels and test its transmission characteristics, as shown by Figure 6b. The measured spectral response closely aligns with our simulated results, exhibiting spectral peak deviations of less than ±2.5 nm and transmission intensity variations within ±15%. By employing a sputtering coating machine with higher fabrication precision, we expect to further reduce these spectral peak deviations to be within ±1.0 nm.

Utilizing the 128-sampling matrix depicted in Figure 6a, we investigate the RS performance by resolving diverse incident spectra at a 30 dB SNR. First, the resolution is verified by conducting dual-peak testing at different wavelength spots, showing a resolution below 0.04 nm. This results in an exceptionally high SPCR of 136.7, which agrees well with our predictions in Figure 2d. In addition, we examine complex sparse or continuous spectra across the entire 700 nm bandwidth, as shown by Figure 6c,d. The results indicate superior reconstruction accuracy with the relative errors *ε* being around 0.01. These findings validate that the designed RS with tailored *ν* will feature a set of significantly improved performance metrics.

## Conclusions

In this paper, we establish a general RS design guideline in accordance with CS theory by revealing the significance of the average mutual correlation coefficient *ν* of sampling matrices. Meanwhile, we propose a universal but powerful RS design with multiresonant cavities. This scheme offers an expansive design space, enabling the system-level optimization of channel spectral responses to facilitate sampling matrices with minimal values of *ν*. Experimentally, we develop a single-shot, dual-band RS on a SiN platform. Photonic crystal nanobeam mirrors are customized to form multicavity channels. As a result, our device achieves an ultrabroad operation bandwidth of 270 nm (covering 1227–1334 nm, and 1453–1616 nm, respectively) along with a <0.5 nm resolution, consuming only 15 sampling channels for each band. This achieves a record-breaking SPCR of 18.0. The temperature tolerance of ±5.0 °C of our device further underscores its design robustness. Furthermore, we illustrate that the proposed scheme can be readily applied to various Photonic platforms. For example, we showcase that an ultrabroadband RS with over 700 nm bandwidth can be created using multilayered optical coatings. Overall, our research presents an innovative solution for developing chip-scale spectrometers with superior performance and may find wider applications in future miniaturized spectroscopic tools.

## Supporting Information Available

Discussions regarding the definition of the mutual correlation coefficient; simulation details for sampling matrices with different values of ν; design of the ultrabroadband Y-splitter; structural parameters of the nanobeams; and fabrication processes of the RS based on multilayer coating (PDF)

## Abbreviations

RS: reconstructive spectrometer
SPCR: spectral-pixel-to-channel ratio
CS: compressive sensing
MEMS: microelectro-mechanical systems
SiN: silicon nitride
PSO: particle swarm optimization
ASE: amplified spontaneous emission
EDFA: erbium-doped fiber amplifier
FDTD: finite-difference time-domain
FWHM: full-width-half-maximums
SOA: semiconductor optical amplifier
SOI: silicon-on-isolator
TiO_2_: titanium dioxide
SiO_2_: silicon dioxide
LPG: long period gratings
DUV: deep ultraviolet
