# Supplementary material for: Benchmarking Reconstructive Spectrometer with Multiresonant Cavities
Source: ACS Photonics. 2024 Aug 15;11(9):3730–40. doi: 10.1021/acsphotonics.4c00915 (PMC11413842; doi:10.1021/acsphotonics.4c00915)
Supplement: Supplementary file 1 — ph4c00915_si_001.pdf [file ph4c00915_si_001.pdf]

Supplementing information

Benchmarking reconstructive spectrometer with multi-resonant cavities

*Chunhui Yao[1], Kangning Xu[2], Tianhua Lin[2], Jie Ma[2], Chumeng Yao[1], Peng Bao[1], Zhitian Shi[1], Richard Penty[1], and Qixiang Cheng[1,2,]**

1. Centre for Photonic Systems, Electrical Engineering Division, Department of Engineering, University of Cambridge, Cambridge, CB3 0FA, UK

2. GlitterinTech Limited, Xuzhou, 221000, China

E-mail: qc223@cam.ac.uk

Number of pages: 4

Number of figures: 1

Number of tables: 1

Section 1

Mathematically, the compressive sensing involves finding an orthonormal basis Ψ to allow that the $\Phi_{N\times 1}$ can be expressed by another vector $a_{N\times 1}$on this basis, which contains only k non-zero elements (i.e. $\Phi_{N\times 1} = \Psi a_{N\times 1}$, as denoted by Eq. (5) in the main text). Consequently, Eq. (4) can be rewritten as:

$$I = S\Psi a = \Theta a$$

The mutual correlation coefficient μ is then defined as:

$$\mu(\Theta) = \max_{i \neq j} \frac{|\langle \Theta_i, \Theta_j \rangle|}{\|\Theta_i\|_2 \|\Theta_j\|_2}$$

where Θ_i and Θ_j are the i-th and j-th column vectors of matrix Θ, respectively. However, for RSs, the continuous nature of channel spectral responses in the wavelength domain results in inevitably large μ values for their transmission matrices, due to the fact that $\Theta_i \approx \Theta_j$, i.e. the adjacent columns always have a large inner product. Therefore, comparing to μ, the average mutual correlation coefficient ν provides a better representation of the overall reconstruction ability over wavelength, which is written as[1]:

$$\nu(\Theta) = \frac{2}{N(N-1)} \sum_{i=1}^{N} \sum_{j=i+1}^{N} \frac{|\langle \Theta_i, \Theta_j \rangle|}{\|\Theta_i\|_2 \|\Theta_j\|_2}$$

Regarding the inverse problem of Eq. (4), i.e. $I_{M\times 1} = S_{M\times N}\Phi_{N\times 1}$, the CS theory reveals that a $S_{M\times N}$ with a smaller value of ν would facilitate a better probability of achieving unbiased estimation of $I_{M\times 1}$[2]. In practice, this translates to a smaller reconstruction error.

Section 2

As elaborated in the main text, we simulate a series of multi-cavity RS and modify the ν of their sampling matrices by adjusting the cavity lengths and the reflectance of mirrors, thereby generating different sampling matrices with the value of ν ranging between approximately 0.4 to 0.9. Here, please note that as we set the channel numbers to 15, 30, and 45, which resulted in matrices of different scales, the optimization of their ν values is actually performed separately using the PSO algorithm. Consequently, the ν values obtained for each of these individual sampling matrices vary slightly, as can be noted in Fig. 2(d) and (f). Nevertheless, this variation does not impact drawing the conclusion that parameter μ/ν serves as a significant benchmark for enhancing the SPCR and reconstruction accuracy of RSs.

Section 3

Figure S1 illustrates the optimization workflow for the ultra-broadband Y-splitter, employing the Lumerical inverse design toolkit[3]. To start, the Y-splitter is modeled as a simple linear taper, with 300 discrete structural points evenly selected along both taper edges as the initial simulation parameters. We then target the transmission efficiency of the fundamental TE mode at both output ports over the wavelength range from 1200 nm to 1700 nm as the figure of merit (FOM) and conduct parametric optimization on all these points. Utilizing a gradient descent approach, this iterative process eventually converges to yield the optimal combination of structural parameters.

To verify its performance, we implement an identical Y-splitter on the same chip as a testing structure. Figure S1(b) displays the measured transmission spectra. As can be seen, across a wavelength range of 1200

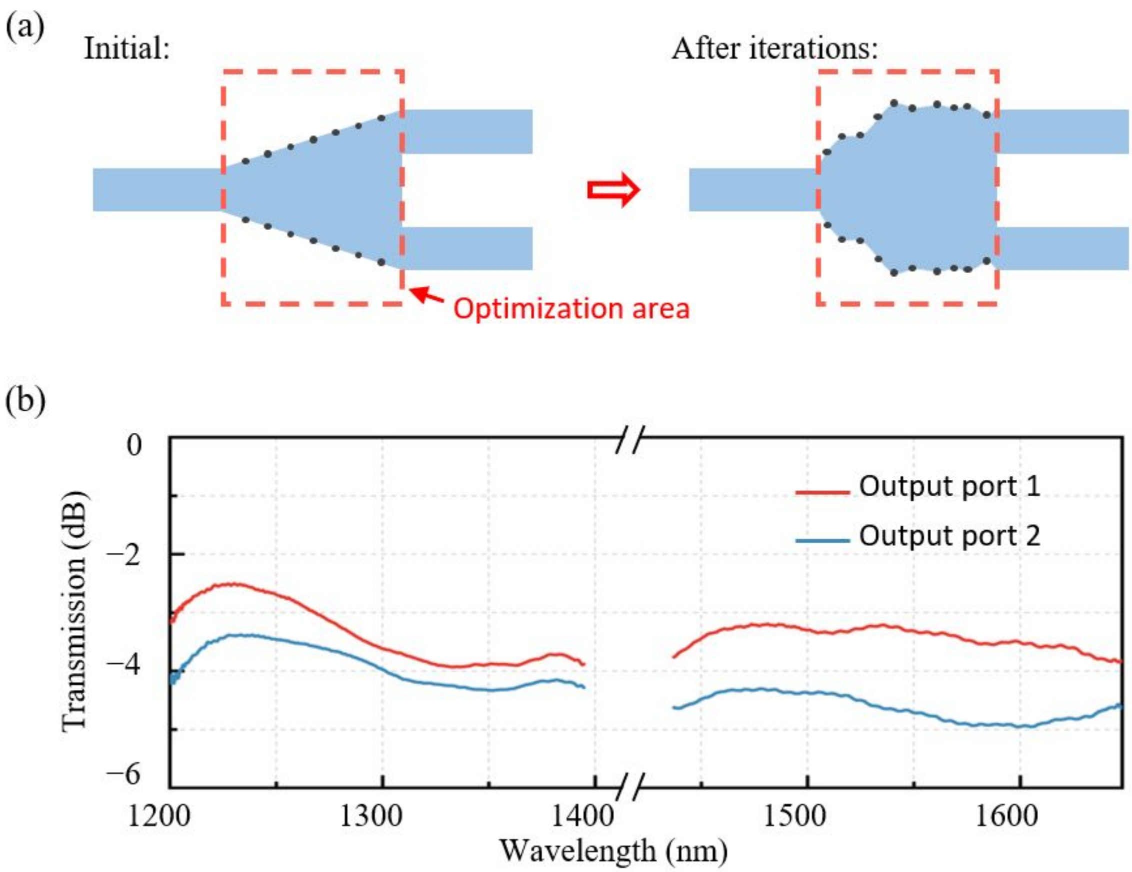

Figure. S1. (a) Schematic of the inverse design process for an ultra-broadband Y-splitter. (b) Measured transmission spectra at the two output ports.

nm to 1600 nm, this Y-splitter demonstrates excess losses of less than 0.9 dB and 1.9 dB at its two output ports, respectively.

Section 4

The optimized geometric parameters for the 1280 nm-band and 1535 nm-band nanobeam mirrors are listed below:

Table S1. Geometric parameters for the nanobeam mirrors

Nanobeam mirrors	Lengths of the major axis	Gaps between etching holes	Waveguide width	Taper width and length
1280 nm-band	T_1 = 320 nm<br>T_2 = 275 nm	G_1 = 785 nm<br>G_2 = 790 nm	W = 1000 nm	W_t= 600 nm<br>L_t= 2000 nm
1535 nm-band	T_1 = 315 nm<br>T_2 = 380 nm<br>T_3= 370 nm	G_1 = 500 nm<br>G_2 = 502 nm<br>G_3 = 500 nm	W = 1020 nm	N.A.

Section 5

To demonstrate the feasibility of constructing high-performance RSs with optical coating technologies, we fabricate and test one specific sampling channel as an example, providing a direct comparison with our simulations. For this fabrication, Schott-D263 glass is selected as the substrate, while an evaporation coating machine are used to alternately deposit 60 layers of TiO_2 and SiO_2 thin films. Considering the limitations of the evaporation coating process and the quality of the coating, the thickness of each SiO_2 layer is maintained between 20 nm and 250 nm, with a total thickness being less than 6000 nm. Similarly, each TiO_2 layer has a thickness between 8 nm and 250 nm, with a total thickness not exceeding 5000 nm

Reference

(1) Bajwa, W. U.; Calderbank, R.; Mixon, D. G. Two Are Better than One: Fundamental Parameters of Frame Coherence. *Applied and Computational Harmonic Analysis* **2012**, *33* (1), 58–78.
(2) Fountoulakis, K.; Gondzio, J.; Zhlobich, P. Matrix-Free Interior Point Method for Compressed Sensing Problems. *Math. Prog. Comp.* **2014**, *6* (1), 1–31.
(3) *Inverse design of y-branch*. Ansys Optics. https://optics.ansys.com/hc/en-us/articles/360042305274-Inverse-design-of-y-branch (accessed 2024-07-30).
